# Managing Information to Improve Conservation: The HBIM of the Wooden Chain of Santa Maria del Fiore

**DOI:** 10.3390/s23104860

**Published:** 2023-05-18

**Authors:** Sofia Celli, Federica Ottoni

**Affiliations:** 1 Department of Architecture and Urban Studies, Politecnico di Milano, 20133 Milano, Italy; 2 Department of Engineering and Architecture, Università degli Studi di Parma, 43124 Parma, Italy

**Keywords:** HBIM, planned conservation, wooden chain, Santa Maria del Fiore

## Abstract

A key potential of HBIM is its ability to reflect the complexity and stratification of built heritage. By gathering in a single place multiple data, the HBIM streamlines the knowledge process that is at the base of conservation activities. By describing the informative tool developed to support the preservation of the chestnut chain of the dome of Santa Maria del Fiore, this paper aims to address the topic of the management of information within the HBIM. In particular, it focuses on how to systematize data in order to facilitate decision making within the frame of a preventive and planned conservation. To this end, the research proposes a possible articulation for the informative apparatus to be associated with the 3D model. More importantly, it makes an attempt to translate qualitative data into numerical values to define a priority index. The latter will improve the scheduling and implementation of maintenance activities, concretely impacting the overall conservation of the object.

## 1. Introduction

When dealing with the conservation of built heritage, the first goal to achieve is gaining a thorough knowledge of the building [1]. This process entails a number of different activities, ranging from historical research to geometric surveys, from visual inspections to diagnostic analyses. This situation calls for a multidisciplinary approach, hence the involvement of many different actors [2]. Although this richness should lead to a comprehensive understanding of the building, it often turns into a weakness. The exchange of information between stakeholders is challenging, as each professional works using their own software and formats [3,4]. The result is a significant communication gap and an unavoidable decrease in the effectiveness of the whole process. The lack of integration between complementary pieces of information hinders the chance of achieving the best possible outcome in terms of both the preservation and enhancement of the architectural heritage. Moreover, due to the absence of a dedicated database, data produced by different actors end up being scattered among different archives and institutions, increasing the risk of their dispersion [5]. Another issue of the traditional conservation approach is that it almost entirely relies on 2D representations. This can be rather constraining given that buildings are actually tridimensional. Even in this case, the risk is for precious information to get lost and forgotten. This generalized unavailability of information may result in an inefficient and costly management of the asset.

While the traditional knowledge process remains essential and irreplaceable, new digital technologies can largely contribute to addressing these issues [6]. By requiring a careful selection and organization of information, they can boost the full understanding of architectural heritage. This becomes even more relevant when acting within the framework of preventive and planned conservation, which efficacy increases together with the knowledge of the building [7]. The more information is gathered, the more accurate the planning of conservation activities is. This will have positive effects on both the preservation and the efficient use of the available resources.

The question of the organization and use of information is thus of primary importance and has been the subject of research for a few years. In fact,

«In a process involving multiple actors, difficulties in communication represent the first obstacle to making the different phases coherent, coordinated and planned. Among the potential advantages of digitization, the facilitation of information management throughout the entire process is perhaps the least obvious and yet the most decisive.»

[8]

To this end, since the 2000s, several information systems have been tested with the aim of systematizing the data collected throughout the knowledge phase. A first prototype developed to support the implementation of conservation plans was named SIRCOP (Regional Information System for Planned Conservation) [7]. The latter then evolved into PlaNet Beni Architettonici [9], which was able to overcome some of the interoperability issues of the previous version [10]. A relevant limit of these solutions was, however, that they used 2D software. The HBIM, working on three dimensions, could hence represent a step forward in the implementation of an informative system that could be used to foster built heritage preservation. 

According to the literature [11,12,13,14], research regarding HBIM has so far focused on geomatic aspects rather than on the organization of information within the model. Indeed, the most relevant challenge when applying the BIM method to historical heritage is the intrinsic rigidity of the modeling tools provided by BIM software. Created to manage the regular shapes of new constructions, they offer a parametric approach that is hardly suitable to portrait the unevenness and deformations of built heritage. Although the need to devote particular attention to geometric modeling is evident—both because of the irregular shape of historic buildings, and because the reliability of the investigations largely depends on the accuracy of the model—it is also true that the absence of a method to guide the filing of information in a result-driven way has now become a criticality. To date, the topic has mostly been addressed in relation to specific case studies [15,16,17,18], and there are no predefined or tested procedures that indicate which data to look for, how to digitize them or how to organize them within the HBIM [19]. The difficulties in retrieving data, their considerable heterogeneity, and the need to take into account the need for continuous updates make any attempt to standardize this process quite challenging. Nonetheless, some considerations in this regard could help the efficient implementation of this promising technology [20,21].

This paper aims to address this question by proposing a possible articulation for the informative apparatus to be associated with the 3D model, showing how new digital technologies can become true operational tools for preservation. To this end, this research focuses on the case study of the wooden chain of the dome of Santa Maria del Fiore, a 24-sided ring hidden between the two shells of the Cupola. Installed between 1423 and 1424 [22], it is one of the least known features of Brunelleschi’s masterpiece. The wooden chain was probably one of the many expedients developed by the designers to overcome the typical collapse mechanism of masonry domes [23], acknowledged on an empirical level [24]. The choice of using wood instead of iron is, however, peculiar, especially if we consider that metal tie rods were commonly used at that time. For this and other reasons, ever since its construction, the device has been the subject of a debate concerning its original role and its current contribution to the monument’s equilibrium.

**Figure 1:**
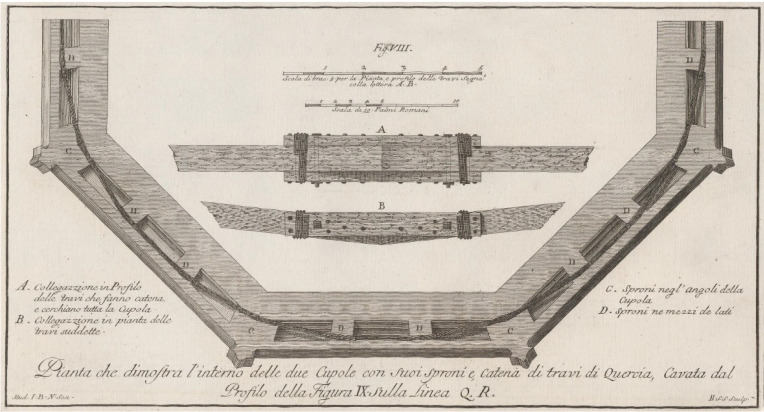
The wooden chain of the dome of S. Maria del Fiore, as surveyed by Giovan Battista Nelli at the end of the XVII century [26].

After six hundred years, the encircling chain still persists and is located about 7.75 m above the base of the dome [25]. While approximating a circle, it is in fact a polygon comprising 24 wooden beams of 30 × 35 cm (w × h) in section, and length ranging between 4 and 5 m. The beams are connected to one another through a system of joints, oak planks and metal elements (Figure 1). 

According to the preliminary analyses performed by the CNR-IVALSA in 2014 [27], the wooden chain still appears to be working, providing a slight contribution to the stability of the dome. Moreover, together with the one located in the baptistery of San Giovanni, the chain is one of the few (if not the only) remaining examples of wooden encircling ties in Italy. This increases its relevance and the urge for it to be preserved.

The HBIM developed within the research aims to provide an easy-to-use tool to ensure the future conservation of this unique artifact. Generally speaking, the attempt is to show how new digital technologies can help address the typical complexity of historical buildings, with positive and tangible effects on their overall preservation.

The paper is structured as follows: Section 2 will illustrate the general background of the research, introducing the methods of the preventive and planned conservation (Section 2.1), as well as the state-of-the-art concerning the discussion on information management (Section 2.2). Particular attention will be paid to the HBIM (Section 2.3), being the technology adopted within the research. 

Section 3 offers an overview on the materials and methods adopted, while Section 4 focuses on the results and contributions of the research. 

The following discussion (Section 5) highlights the limits and strengths of the proposed solution, indicating the possible future steps of implementation. Finally, in the conclusions (Section 6), the results are reconsidered in a broader context, bringing the focus back to conservation and underlining how new digital technologies could and should be exploited in order to improve the preservation of architectural heritage.

## 2. Background

### 2.1. Preventive and Planned Conservation

The concept of Programmed Conservation, introduced in the 1970s by Cesare Brandi as “Programmed Restoration” [28] and later refined by Giovanni Urbani [29], refers to a strategy based on risk assessment and control. Through periodic inspections and reiterated minor repairs [7], this strategy aims to limit the incidence of restorations as traditionally intended, thus fostering material permanence. The underlying idea—already expressed in extreme terms by Ruskin [30]—is that by providing constant care, it is possible to avoid—or at least postpone—invasive interventions and replacements, which inevitably impact the building’s authenticity and, consequently, its value. This is, simultaneously, the main strength and weakness of this strategy. The purpose of preservation should be to maximize permanence while cutting costs. Nonetheless, this approach is not so frequent, and cases in which a planned conservation strategy has actually been adopted are particularly virtuous [31,32]. Indeed, while still requiring consistent resources, the outcomes of preventive conservation are not immediately perceivable. Due to a substantial lack of funds, those who sponsor restoration interventions, however, want the results to be evident and instant. Although things are slowly changing, this situation ends up limiting the diffusion of this valuable approach. 

Today, Programmed Conservation can be described as follows:

“a medium-long term strategy for the effective management of cultural heritage. Based on the integration of conservation and enhancement activities. It is oriented towards prevention and the constant care of cultural heritage. It is an articulated process which produces new knowledge and stratification of information, and requires tools for data management and planning.”

[33]

The definition reflects the complexity of the task, which requires a broad and dynamic vision. It also highlights the need for regular inspections, which make it possible to monitor the state of repair of the individual elements, to verify the efficacy of any implemented preventive measures and, finally, to efficiently schedule further conservation activities [34]. Within the framework of planned conservation, defining a frequency of inspection becomes a critical matter [35]. Unlike new buildings, where the useful life cycle can be reasonably estimated, the “durability” of historic architecture is difficult to standardize. In the absence of reliable experimental data to refer to, inspection frequency will have to be evaluated from time to time, following the principles of “graduality” (progressive evaluation of the severity and speed of evolution of the processes in progress), “flexibility” (possibility of reassessing the frequency of inspection if necessary) and “opportunity” (chance to optimize available resources by combining different inspections together). The chronology of the interventions is thus an indispensable tool in order to continuously improve the forecasts and, consequently, the scheduling of conservation activities [36]. It goes without saying that this whole process (continuously reiterated) produces a huge amount of data that require proper archiving and management tools. 

### 2.2. The Issue of Information Management

When dealing with the issue of information management, a premise is needed: a greater stratification of the analyzed building will entail a greater stratification of the collected information. Thus, when operating on historical heritage, one must take into account that the amount of data will be considerable and their management rather complex, also by virtue of the fact that stand-alone information can hardly be significant. Indeed, to fully understand the reality, it is necessary to correlate information and analyze it critically. Only in this way will it be possible to generate knowledge or, at least, the specific knowledge upon which conservation activities should be based.

The question of data archiving hence needs to be better clarified: the aim is not to indiscriminately collect data, but to make a considered selection of the information that will truly result in a deeper knowledge of the object, thus improving its management [37]. Therefore, the primary goal must be to offer an informative system that supports both the execution of conservation activities and their planning [38]. 

Before computers, knowledge was passed on via paper support, the finiteness of which necessarily required summarization [39]. Today, thanks to new technologies of unimaginable potential, we could ideally record an infinite amount of information. Within the construction industry, the most promising method is the one proposed by the Building Information Modeling (BIM), which proposes the use of Digital Twins [40] as tools by which to implement predictive management. While this approach seems particularly fitting for new constructions, its fruitful applicability to built heritage is still being questioned. Considering the thorough analyses and careful interpretations needed to fully understand a historical building, will the virtual model be able to retain the stratification and complexity of a monument? For the moment, the answer would seem to be no—or at least not entirely. This does not mean that new digital tools should not be explored and exploited to their full potential, bearing in mind that any ITC tool developed for the purpose of heritage conservation must have as its primary objective an adequate transmission of knowledge [38]. However, the aim of this research was not to delve into the functioning of data management software on a technical level, but rather to focus on the contents and their organization. It is therefore a question of examining and improving the process that leads from knowledge to information. The information system [41] must grow into a decision-making and operational support for setting up, implementing and managing maintenance activities [42].

A well-structured information system can facilitate the dialogue between professionals who have different skills and languages. In this specific field, besides the actors traditionally involved in architectural design, there are other professionals (surveyors, historians, diagnosticians, etc.) who will need to communicate, share information and, finally, offer their own contribution to the knowledge of the building. Nonetheless, on a practical level, professionals operating in the sector often lack the specific skills required to efficiently use complex models, and only partly exploit their potential [43]. A greater involvement of restorers in the development of the informative tools is thus crucial, as they themselves are to be the first users. The organization of data and their transmission through the informative model are the result of critical choices, which can influence the final outcome. An altered knowledge process can thus lead to an inappropriate conservation strategy [37].

A few scholars have developed some interesting considerations in this regard. While referring specifically to the field of structural evaluations, they point out how a perfect coherence between the real object and its virtual counterpart is not a guarantee of the correct interpretation of the ongoing mechanisms. Sometimes, the similarity between model and reality must be mitigated in order to truly grasp the etiology of the instability mechanisms without distractions. It hence becomes necessary to operate simplifications aimed at reducing the accuracy by selecting only part of the potentially available information [44].

We could conclude that the continuous refinement of the available software in terms of accuracy—and therefore the gradual increase in stored data both in terms of quantity and quality—does not necessarily represent the solution to the problem [19]. The level of detail and amount of information must in fact be oriented to the final result.

The need to make critical choices hence emerges again, highlighting once more that, within the context of built heritage preservation, traditional practices have not been completely replaced by digital technologies. Nonetheless, it is evident that the informative tools currently used in the construction industry are an unmissable opportunity which, in the long run, will lead to an easier and more coordinated management of historic buildings.

### 2.3. HBIM as a Possible Solution

In recent years, a new methodology has made its appearance in the world of architectural design: the BIM (Building Information Modeling). The latter aims to improve the management of buildings throughout their whole life, from design to construction and maintenance. The particularity of this technology is that it integrates traditional design tools (2D drawings and 3D models) with other heterogeneous data concerning the entire life cycle of the building. On the one hand, this facilitates managerial aspects, and on the other, it fosters communication between the actors involved in the design and construction phases [45]. Basically, BIM is the digital transposition of the physical and functional features of an object.

The enormous potential of this tool in the context of new buildings has prompted restorers to verify whether it can also be fruitfully applied to built heritage. Although differences in purpose may discourage the export of this methodology to historical buildings, some of the possibilities it offers could greatly facilitate conservation practices. The documentary aspect is particularly appealing: the opportunity to associate data with individual 3D construction elements makes the BIM consistent with the objectives of the information tool described in the previous paragraph.

The development of HBIM (Historical or Heritage BIM) [46,47] has been fostered by the considerable progress made in recent years in the field of geomatics. Another important factor is the increasing diffusion of new conservation approaches which, more and more frequently, favor planned conservation over traditional restoration [48]. Within this framework, informative aspects have gained renewed importance, and the development of long-term management strategies has become critical. The HBIM aims to join these two aspects, bringing together the opportunities offered by geomatics and the principles of planned conservation. In this sense, the potential offered by HBIM can be summarized as follows:

- Documentation/knowledge—the HBIM facilitates the collection of heterogeneous information, such as data concerning construction and maintenance history, as well as the geometric, functional, structural and material characteristics of the building and its individual elements.
- Ease of management—the model will support conservation activities by facilitating the planning of interventions. The information system will be constantly fed new data generated by periodic inspections, gradually increasing its forecasting potential.
- Sharing—the tool will foster cooperation among the professionals involved, ensuring clearer communication and greater consistency between the results. The same model can in fact be used to carry out analyses of different kinds.
- Enhancement—the model could be easily implemented using virtual or augmented reality technologies, with possible interesting implications in terms of dissemination.

Despite these obvious advantages, the HBIM has only recently found application in Italy. In the last five years, some standards have finally been issued [49,50]. Such standards, however, refer to BIM methodology in general, rather than to HBIM specifically. The novelty of HBIM makes it an interesting research topic. The main question being addressed is the possibility of modeling complex geometries in BIM environments [11,12,13,14]. Indeed, the latter show some rigidities owing to the fact that BIM was originally intended for new constructions and did not take into account the need to model irregular or deformed shapes. A few studies, among which is the one here presented, are finally working on the management and systematization of information within the HBIM, with the purpose of actively support heritage preservation [6,15,16,17,18,19].

## 3. Materials and Methods

**Figure 2:**
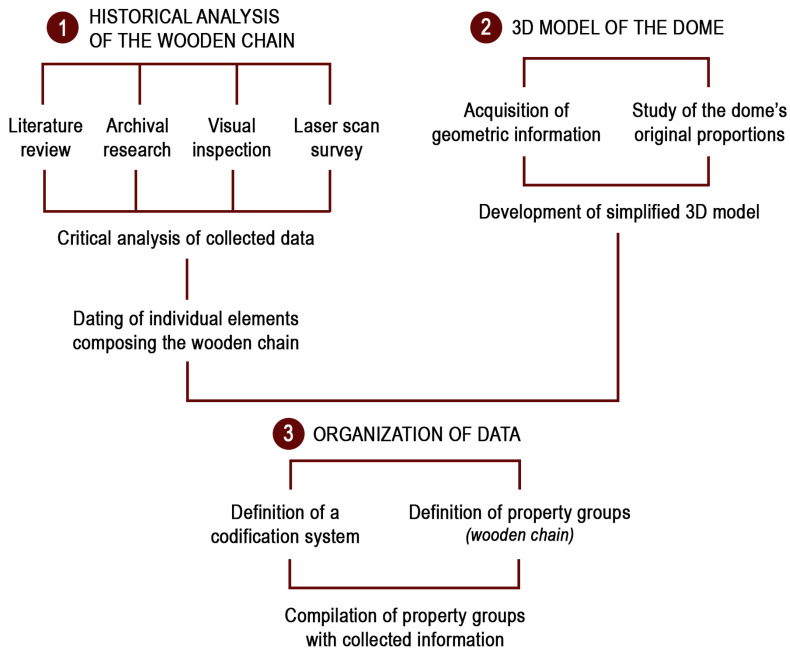
Schematic overview of the adopted methodology for the implementation of the HBIM.

The research combines the H-BIM methodology with the preventive and planned conservation approach. Within this framework, the focus has been set on the organization of data within the H-BIM, which is currently by no means standardized. The paper thus aims to contribute to the development of H-BIM on the information side, rather than on the geometric and modelling side. To this end, the research has been articulated in different phases (Figure 2):

- Historical analysis of the wooden chain: the construction history of the wooden chain has been retraced thanks to a thorough study of archival sources and literature, to visual inspections and to an accurate geometric survey. This phase generated lots of data which needed to be catalogued and made accessible in order to foster and improve conservation procedures.
- 3D model of the dome: starting from 2D drawings obtained from two different surveys of the dome, a simplified 3D model of the dome and wooden chain was developed. The model aims to represent the building in its original, undeformed situation. To this end, the simplification of the model has also entailed the study of the original proportions of the dome.
- Organization of data: in the last phase data collected throughout the research have been organized into property groups to be associated to the 3D model in BIM environment. As explained in the following paragraphs, data have been organized in order to provide the most possible benefits to the professionals in charge of the preservation of the wooden chain.

### 3.1. Historical Analysis of the Wooden Chain

Despite the relevance of the wooden chain and the long-lasting debate concerning its original and current role, a systematic study of this device had not been carried out up to now. Hence, starting from the countless studies on Brunelleschi’s iconic dome [22,24,25,51,52,53,54,55,56,57] (including a few preliminary studies on the chestnut chain [23,27,58,59,60,61,62]), a historical analysis was started. The aim was to retrace the construction and maintenance history of the wooden device. The objective of the research was to clarify the original configuration of the encircling tie and to establish the efforts—either regular or sporadic—made to ensure its conservation over the centuries. To this end, the information that emerged from the literature and archival documents was combined and critically analyzed. The outcomes of the research were then intertwined with data coming from other sources, such as the precision geometric survey carried out in 2018 by studio Scaletti and studio COMES, direct observations and historical treatises. Through this process, a plausible dating for each of the elements comprising the wooden chain was proposed [63]. Specifically, it was found that the original metal joints are still the most widespread today, while the other joint typologies are attributable to successive additions or replacements. All of them were supposedly installed before 1848, when, according to archival documents, the last maintenance intervention took place. Throughout the 20th century, the wooden device did not receive any care and different studies [52,54,64], suggest that, at the time, the general belief was that the chain did not have a relevant role in the stability of the dome.

The large amount of data collected during the research and the need to reinstate maintenance practices for the wooden chain has fostered the development of an information tool. The latter has thus become the means by which data (historical and non-historical) might find operational value, confirming the essential role of knowledge within the framework of built heritage preservation.

### 3.2. 3D Model of the Dome

After examining the context, and understanding the objectives to be achieved according to the needs of the Opera di Santa Maria del Fiore, the research moved towards the organization of the information tool. The first step was the development of the three-dimensional model of the Dome, created using McNeel’s software Rhinoceros. As mentioned, one of the most critical issues of the HBIM methodology is the difficulty of faithfully reproducing geometries. The shape of historic buildings, as it appears today, is the result of centuries of transformations and stratifications, owed to aging, structural deformations, traumatic events, and changes in use. These specificities cannot be overlooked when aiming to fully understand the architectural object in its historical, formal and structural aspects. Nonetheless, they represent a significant obstacle when trying to use the digital twin technology. Indeed, the creation of a three-dimensional model that is completely consistent with the reality involves important investments in terms of time and money. Moreover, the results, although extremely accurate, incur the risk of not being entirely appreciable due to the excessive size of the files produced [4]. More importantly, they might not be so effective when assessing structural conditions.

In many cases, the “actual” geometry is not the most useful to model, whereas the “reference” geometry of the building, in its undeformed state, can be more reliably analyzed to highlight the actual deformations of the structure. This is not an easy task because it requires the integration of different data, such as the theoretical geometries that were used in the past, the constructive geometries (related to different building techniques), and the geometrical variations, owing to changes in use or restoration works. By verifying the geometrical compatibility between the deformed geometry of the structure and the hypothesized damage mechanisms, it is possible to identify the actual damage mechanisms. This can be achieved by applying the gravitational loads and, if necessary, a set of displacements related to the identified mechanisms [65]. In this process, the hypothesized mechanisms can be validated by comparing the deformed geometry of the structure, obtained using the structural analyses, with the “actual” geometry of the structure [66].

**Figure 3:**
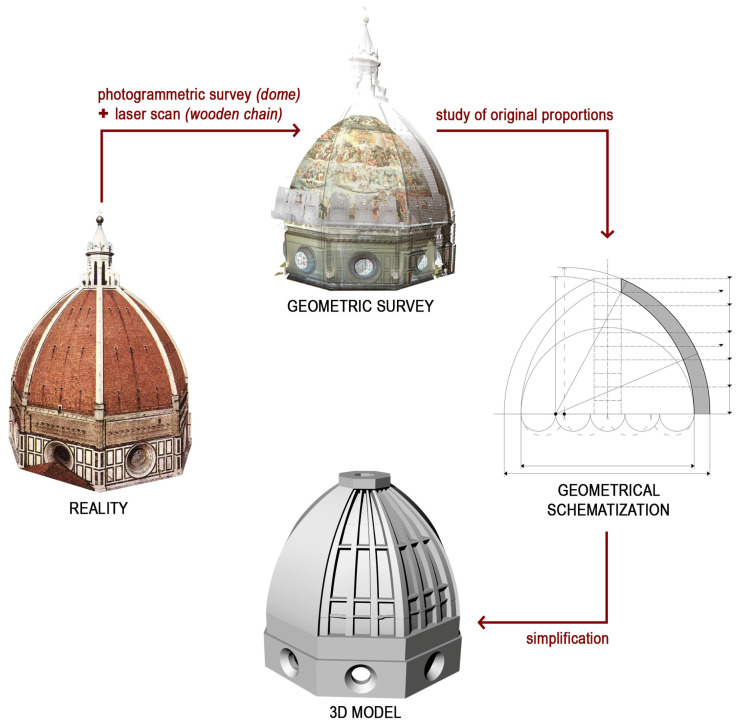
Diagram showing the process of geometrical simplification of the 3D model.

Starting from these assumptions, the modeling of the dome of Santa Maria del Fiore was based on geometric simplifications and regularizations (Figure 3). The primary objective of the information tool hereby described is not to faithfully reproduce the geometry of the building, but to maintain an adequate level of detail in order to distinguish the individual elements comprising the dome, so as to be able to systematically associate the available data. According to the AIA G202-2013 protocol, the level of development of the model could be associated with a LoD 200. A distinction between the LoG (level of geometry) and LoI (level of information) should however be made; while the LoG is approximate, the LoI is far more precise, possibly reaching a level 400. On the one hand, the different elements comprising the model are not completely coherent with reality in terms of their dimensions and geometry. On the other hand, the large amount of information collected on the wooden chain during the research have been associated with the individual objects within the model that increase the LoI. Despite the low geometric accuracy of the model, the possibility of geo-referencing the information will facilitate its consultation. This will increase the knowledge of the building, thus resulting in the more accurate planning of inspection and maintenance activities.

The model was built according to geometric and metric information collected from two different surveys. The first one concerns the entire dome and was carried out between 1992 and 1994 by FO.A.R.T. s.r.l. using the photogrammetric method [67]. The survey was performed on the occasion of the restoration of the dome, taking advantage of the scaffoldings. Measurements were acquired using a theodolite Wild T2000S. To solve the problems related to the narrowness of the spaces and the scarce stability of the topographic stations, the Wild mini RMS system was used. While this survey is not the most recent or advanced survey of the dome, it is the only one that has been published [67] and was thus available to the authors. The second survey used to develop the 3D model was the laser scanner survey performed on the wooden chain in 2018 by Studio Scaletti and Studio Comes. A Faro Focus 3dS 120 scanner was used on this occasion, for a total of 208 points of TLS acquisition. Given the total length of the wooden chain (about 150 m), to limit the overall error, a topographic survey was also carried out using a Geomax Zoom 90 total station (15 topographic vertices, and 93 control points for the control and optimization of the different cloud registering). The survey was particularly problematic because of the position of the chain. The latter is located about 5 m above the ground, in the dark cavity between the two shells of the dome. To overcome these issues, an extensible tripod was employed and scans were acquired in grey scale. The laser scan survey is the only complete survey of the wooden chain. Before 2018, the existing representations of this object were based on the drawings made by Giovan Battista Nelli at the end of the XVII century (Figure 1).

**Figure 4:**
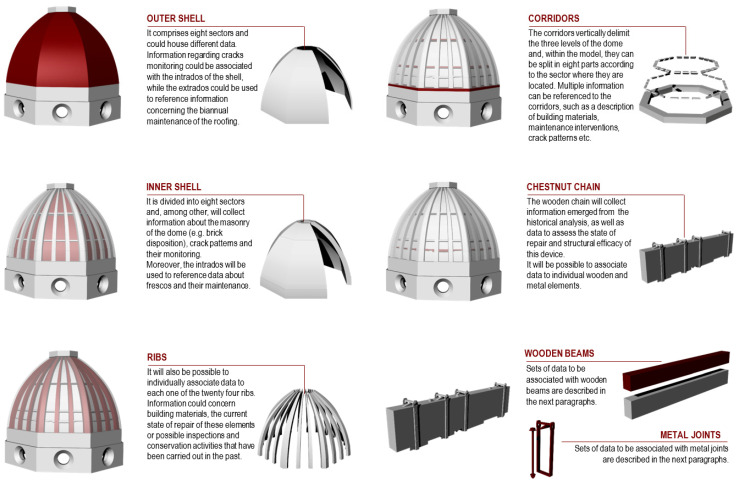
Identification of the different construction elements comprising the 3D model.

The 3D model of the dome was developed using the 2D drawings provided by the mentioned surveys. Aiming to represent the undeformed and simplified geometry of the building, the current geometry of the dome was further schematized, based on the study of the original design proportions of the dome. The lengths of the eight sides of the drum were assumed to be equal, just as the rib and shell sectors were all modelled using the same curvature and tapering. As for the wooden chain, the beams are represented as the regular elements of a rectangular section and the metal joints are regularized as well. Despite its abstraction, the resulting model comprises all the main construction elements that compose the Dome (Figure 4). 

#### The Codification System

A codification system was developed in order to univocally name each of the elements comprising the dome. According to the indications provided by the 2003 guidelines [68], a set of alphanumeric codes was created. The latter refer, on the one hand, to the typology of the technological elements (cap, rib, wooden chain, etc.) and, on the other hand, to their location within the dome (sector, room, level, etc.). In this way, the operators in charge of maintenance activities will be able to easily identify the different elements and name them unequivocally within the produced documentation (reports, graphs, photographs, etc.). Clearly, the same classification will be attributed to the objects within the information model. The structure of the codes is illustrated in Table 1. The latter only shows codes related to sector 1, which serves as an example. The coding process will, however, be repeated in the same way for each of the eight sectors of the Dome.

### 3.3. Organization of Data

Before effectively starting to catalogue and organize data, the main purposes of the information tool were identified, in order to choose the most suitable software for the implementation of the HBIM:

- to offer the technical office of the *Opera di Santa Maria del Fiore* an intuitive and easy-to-use tool to support conservation activities;
- to develop an effective cataloging system for the archiving and retrieval of information;
- to foster collaboration between the professionals involved in the management of the Opera’s assets;
- to guarantee the usability of the information tool on a large scale (possibly also for dissemination purposes).

On the basis of these considerations, it was decided that commercial software would be opted for, which, although intended for new buildings, are more widespread and known. The advantage is twofold: these software are easier to acquire and to use, given the numerous courses usually offered by the software companies. Since the 3D model was developed using Rhinoceros, it was decided that the HBIM would be developed using Archicad (Graphisoft), in order to benefit from the Grasshopper-Archicad Live Connection. A further influencing factor was the BIMx application offered by Graphisoft, which guarantees access to the information model from mobile devices such as smartphones and tablets. This opportunity is particularly interesting, especially since it will allow professionals to have direct access to much useful information directly on site. This will favor the accuracy and correct execution of the planned operations.

Finally, the information was organized and structured. Useful data were identified, grouped and arranged in an intelligible way, to make them easily available to those who will be in charge of conservation activities. This operation was carried out for both wooden elements and metal joints. While the wooden chain was used as a pretext for illustrating the potential of the HBIM, the ultimate goal envisages the extension of the proposed system to the whole dome.

**Figure 5:**
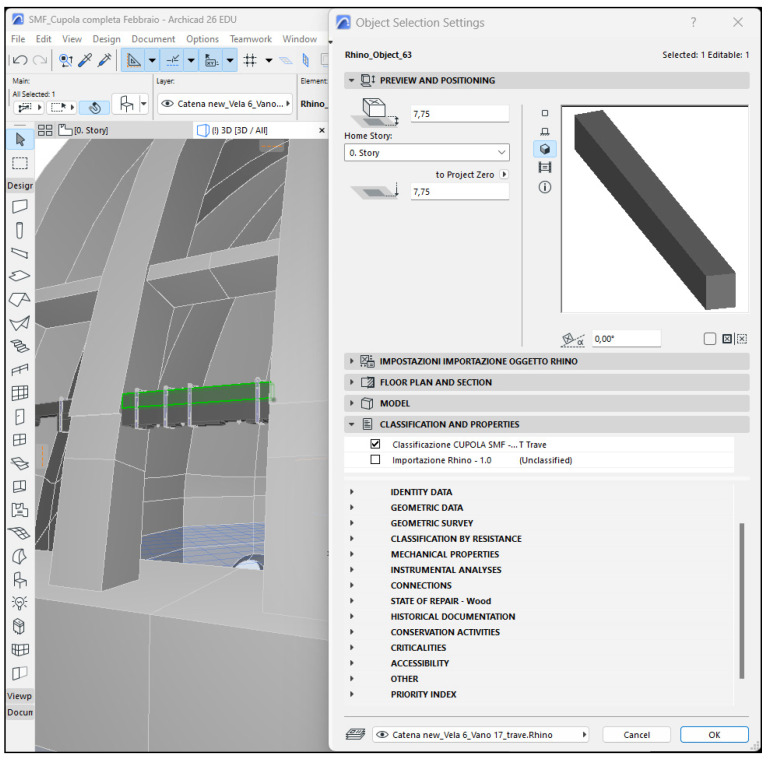
The image shows the complete list of property groups used to describe wooden elements.

Before starting to structure the information system, a few pertinent references were consulted, with particular reference to the 2003 guidelines [69], the UNI 11257:2007 standard [70], and a book by Cecchi and Gasparoli [42] concerning procedures for inspection activities. Standards specifically dedicated to cultural heritage were also considered [71,72,73], as well as information gathered during the previous historical–critical investigation of the wooden chain. To effectively organize information regarding the wooden beams, a few complementary references dealing specifically with wood were taken into account. The UNI 11119:2004 standard [71], dedicated to in situ inspection of wooden elements, was particularly suitable, because it is explicitly dedicated to cultural heritage. This means that all the operations suggested within the norm take into consideration typical conservation instances (for example, the use of non-invasive diagnostics). Further useful suggestions were retrieved from the preliminary report by the CNR IVALSA laboratory after performing some sample tests on the beams of the wooden chain [27]. The aim of the analyses was to determine the wood species, the state of conservation and the state of tension in the beams. Starting with these indications, the different groups of properties to be attributed to the chain’s wooden elements were organized (Figure 5). 

**Figure 6:**
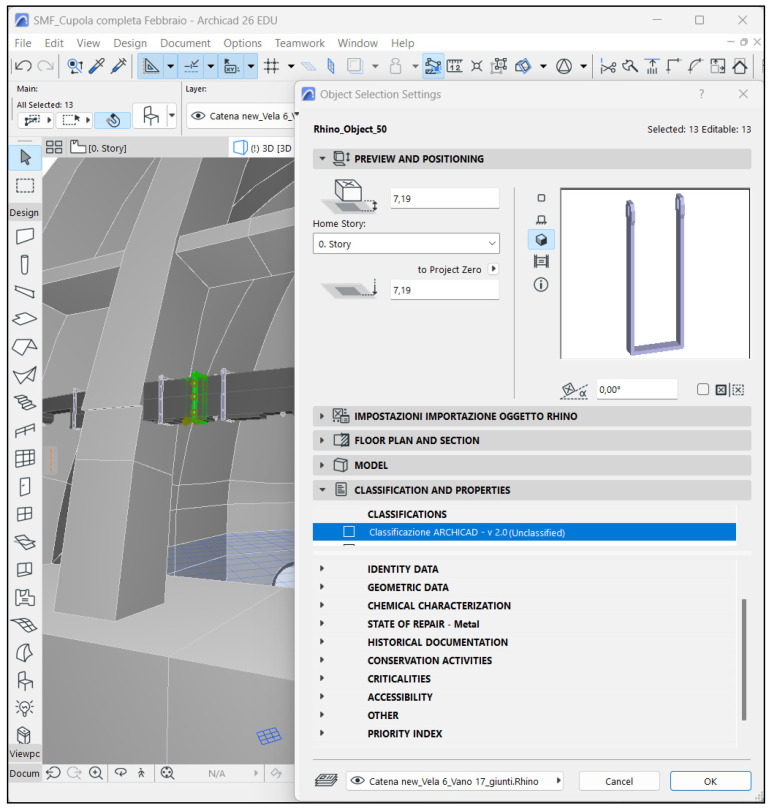
Overview of the groups of properties that were set up to describe the metal joints.

The information system developed for metal joints is largely analogous to the one for wooden elements (Figure 6). Although the general scheme is unchanged, given the differences between the two types of objects—in terms of materials, form and function—some necessary adjustments were made. In particular, the sections “classification by resistance”, “mechanical characteristics” and “connections” were omitted, while the “identity data”, “geometric data” and “state of repair” groups were amended. Without having identified standards that are specifically dedicated to the evaluation of the conditions of historical metals, the organization of the data was defined on the basis of the information collected during the historical–critical investigation and in accordance with the 2003 guidelines [69].

## 4. Results

The following subparagraphs illustrate the property groups that have been structured to archive the data concerning the wooden elements comprising the encircling tie of the dome of Santa Maria del Fiore. This sorting of information represents the most relevant result of the research, as it proposes a way to organize data concerning wooden elements in a preservation-oriented way. The same cataloguing system, with a few due adjustments, could be applied to other case studies dealing with wooden structures. Each property group is thus thoroughly described and explained, also highlighting possible concrete effects on conservation processes.

### 4.1. Identity Data

**Figure 7:**
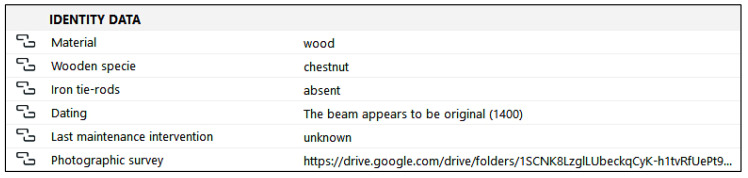
The identity data property group. The information to be provided include the type of material used and the specification of the wood species, the presence or absence of strengthening iron ties, the hypothesized dating of the element, and the year and kind of last maintenance intervention. Photos of the element can also be accessed via the hypertext link.

The first property group includes the most relevant data for the identification of the specific element (Figure 7). It could be seen as a summary of the more detailed data offered by all the other property groups. In particular, the group includes a field indicating the element’s material and, since we are dealing with wooden beams, a further specification has been provided regarding the wood’s species. The next entry concerns the age of the element (year of installation); the datum to be inserted is set as a text string, in order to allow the compiler to annotate any uncertainties. It is then requested that whether there are strengthening elements is indicated, with specific reference to the iron tie rods that were diffusedly added to the wooden chain. Indeed, the presence of metal tie rods greatly influences the strength of the beams and must therefore be taken into consideration when defining intervention priorities. While in this property group it is only possible to indicate whether iron tie rods are present or absent, further information concerning these elements will be provided when describing the tie rod itself.

The identity data also include an entry concerning the last maintenance intervention (date and type of activity) and, finally, a photographic survey, which will be associated with the model through a hypertext link.

### 4.2. Geometric Data

**Figure 8:**
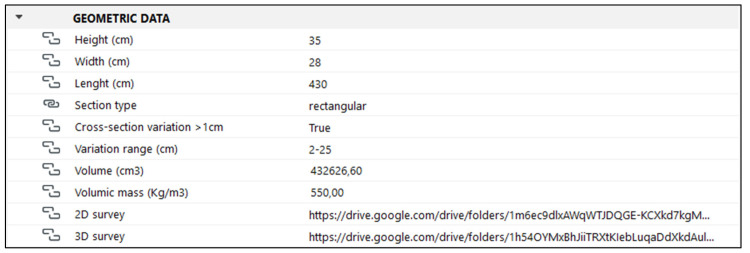
Overview of the geometric data property group, which offers detailed information about the geometry and dimensions of the object. The 2D drawings and point clouds can also be accessed via hypertext link.

The group of properties dedicated to geometric data (Figure 8) is of fundamental importance, especially since the 3D model—representing an undeformed and regularized object—is not entirely coherent with the reality. This inconsistency is partly overcome thanks to this property group, which offers precise data gathered from the recent laser scanner survey. In addition to metric data regarding the main dimensions of the wooden elements (length and section), it will be possible to include, in this group, 2D drawings as well as the point cloud. The use of hypertext links will thus enable high-precision geometric information to be made available without increasing the size of the model.

As for the length of the beams, it should be mentioned that the provided dimensions only refer to their visible portion, as it was not possible to measure to what extent the extremities enter the masonry ribs of the dome. In addition to linear dimensions, the volume and density of the wooden elements are indicated, according to the UNI 11035-2 standard [74].

Finally, three entries have been dedicated to the section of the beam, providing specifications concerning its geometry and possible variations. In particular, with regard to the type of section, an option group has been preset, so that it will only be possible to select an option among the following alternatives: rectangular, square, round, beveled and irregular. On the other hand, for variations in section, a true/false option was set. If there are relevant variations (true), it will also be requested that the variation range is indicated.

### 4.3. Geometric Survey

A further property group is dedicated to the geometric survey, in which details concerning the source of the geometric data will be provided. The group comprises four items: the first contains the names of authors, the second indicates when (year) the activities were carried out, the third shows the brand and model of the equipment that was employed and the fourth gives access to the eventual reports via a hypertext link.

### 4.4. Mechanical Properties

**Figure 9:**
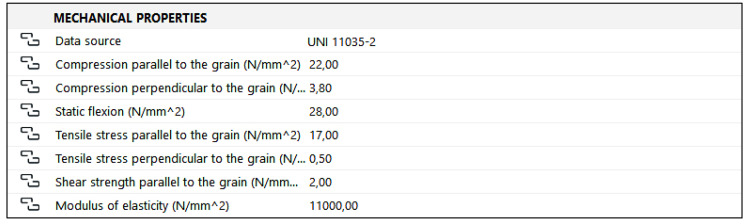
Structure of the property group concerning the mechanical properties of wooden elements. The listed properties have been selected according to the UNI 11035:2010 standard [72] on structural timber.

This property group is meant to collect information concerning the mechanical characteristics of the material (Figure 9), in this case, of different wood species. The first item specifies the origin of the data, which can be selected from an option group including the main sources providing this type of information.

In particular, the following sources were considered:

- any instrumental or laboratory tests;
- the UNI 11119:2004 standard [71]—which offers indications of the procedures used to perform the on-site assessment of the strength and stiffness of timber elements, with specific reference to the field of cultural heritage;
- the UNI 11035:2010 standard [74]—which, in Part 2, provides the typical mechanical properties of the main Italian wood species.

Each of these options corresponds to a different level of knowledge. While in the case of instrumental testing, the data accurately reflect the characteristics of the specific object, in that of visual classification [71], considerable approximations are made. However, the values that are the most generic are clearly those derived from the UNI 11035 standard [74], as they are «typical values», which do not take into account the physical conditions of the element nor its surrounding context. The UNI11119 [71] classification is a rather good compromise; although it still proposes typical values, it makes an attempt to adapt them to the specific situation by proposing coefficients of correction. The classification is determined according to the results of visual inspections and non-invasive analysis, which is particularly appreciable when dealing with cultural heritage. Ultimately, the operator who will have to fill in the database, in the absence of precise data, will be able to rely on the values proposed by the UNI 11035 standard [74]. In this case, it is, however, recommended that inspections and/or tests are planned in order to obtain a more precise characterization of the material properties.

### 4.5. Classification by Resistance

To facilitate the visual classification of the wooden elements proposed by the UNI 11119:2004 standard [71], a further property group was developed. According to the standard, the first thing to do in order to classify a wooden element is to assign it to a “category” (I, II, III) on the basis of its specific features, including its alterations and particular defects. Each category indicates different mechanical properties, and the UNI standard sets the range of values that determine to which category the timber element needs to be assigned. For the sake of clarity, the technical or equivocal terms are defined at the beginning of the document, in a dedicated paragraph. Furthermore, where needed, the standard specifies the measurement procedures to be followed. This occurs, in particular, in the case of chamfers, individual nodes, groups of nodes and the slope of the grain.

**Figure 10:**
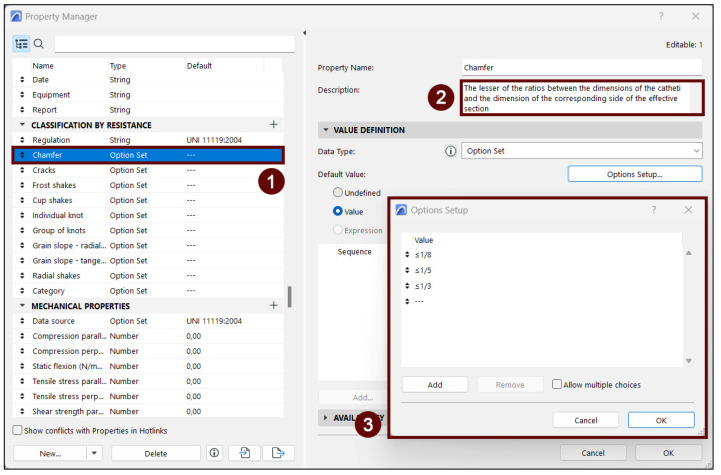
The image shows how the classification by resistance property group was set up. The aim of the group is to guide operators through the on-site assessment of timber elements. To this end, each item (1) was associated with a precise description of the requested data (2). Moreover, an option set (3) was pre-compiled using the threshold values specified by the UNI 11119:2004 standard [71]. In this way, the operator will only have to pick one out of the list.

In order to simplify the compilation of the property group, these indications are also displayed in the HBIM model, within the description field of the individual properties (Figure 10, n° 2). In this way, the operator can easily proceed with the on-site assessment without having to resort to the full text of the standard. For the same purpose, it was decided that the values for each property would be pre-set, using an option group indicating the thresholds provided by the standard (Figure 10, n° 3). Finally, it should be noted that the efficacy of this method of classification can be influenced by the accessibility and visibility of the analyzed elements. When dealing with timber elements that are not fully visible, non-destructive instrumental tests will be needed to make up for the lack of information.

### 4.6. Instrumental Analyses

The property group developed to collect data regarding the possible instrumental analyses is partly fixed and partly variable, in order to embrace the specificities of the different types of analyses. In particular, entries referring to legislation, author, date, instruments, testing location, report are considered unchangeable. The remaining items can be modified according to the specific results. The need to provide a certain level of flexibility is owing to the heterogeneous nature of the tests’ outcomes.

The idea is therefore that, each time an instrumental test is performed, the property group can be duplicated and partly adapted to specific needs, while keeping the abovementioned fields unchanged. The latter are in fact essential in order to guarantee the traceability of the procedures, which is the basis of a correct scientific investigation. In particular, the reference to the legislation will allow one to check the method that was used to carry out the tests, partly overcoming the possible lack of descriptive reports.

The identification of the equipment is equally important, as it enables the level of accuracy of the investigations to be assessed on the basis of the technical specifications. Similarly, the indication of the date of the tests will help determine their current reliability. Finally, the reference to the location of the tests is fundamental, because it will potentially enable the results to better correlate with one another, as well as with the entire object and its context.

### 4.7. Connections

Given the large number of both wooden and metal joints on the chain, a section of the informative tool has been dedicated to the connections. Within this property group, three entries concern wooden joints and the other four concern metal joints. A final entry summarizes the total quantity of connections identified on the beam. For both categories of joints, the following information is requested:

- presence/absence—an option group that indicates whether any joints can be seen on the wooden element.
- typology—a brief description of the joints is provided. While a text string was arranged for wooden joints, for metal joints it was decided that a preset option group would be used; this includes the six typologies that currently exist on the wooden chain. An additional field is created for metal joints, in order to provide access, via a hypertext link, to a more detailed description of the specific joint typology.
- quantity—the number of joints of each category (wood and metal) will be provided. Where no joints are found, a 0 value will be indicated.

### 4.8. State of Repair

**Figure 11:**
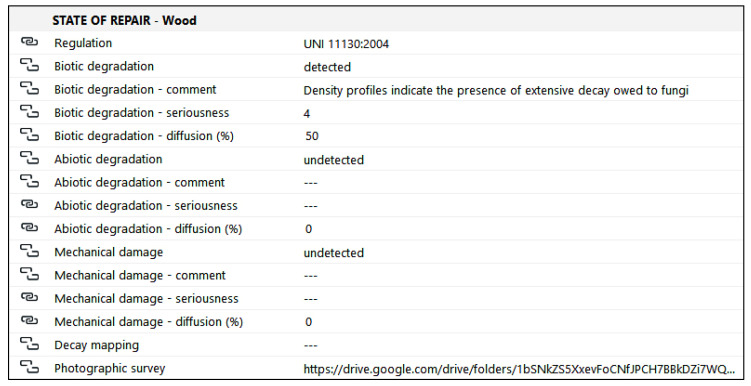
Overview of the group of properties describing the state of repair of wooden elements. According to the UNI 11130:2004 standard [72], three types of decay are considered: biotic degradation, abiotic degradation, mechanical damage. For each one of them, the property group requires that, if detected, a comment is provided, and the seriousness and diffusion of the phenomenon is evaluated. Thematic maps and photographs of the decays can be accessed via hypertext link.

A group dedicated to the description of the state of repair is also arranged (Figure 11). The first request is to indicate which standard was used (if any). In this case, we followed the UNI 11130:2004 [72], which offers terms and definitions concerning wood deterioration. The following main decay phenomena were identified accordingly:

- biotic degradation—form of degradation caused by biological agents (fungi and insects);
- abiotic degradation—form of degradation caused by both chemical and physical agents;
- mechanical damage—mechanical failure caused by internal or external mechanical actions.

By re-elaborating the description of decay proposed by the 2003 guidelines on maintenance plans [69], four entries were created for each macro category of deterioration: deterioration (type), comment, seriousness and diffusion. The first item is named after the specific category of decay (e.g., biotic degradation) and whether that specific decay can be found on the analyzed wooden element to be indicated. Its presence or absence is expressed through an option group (detected/not detected). If the problem has not been detected, the additional items referring to the specific category of decay will be left empty. Otherwise, a brief comment will have to be added to better clarify the nature of the deterioration, providing a qualitative description and, when possible, highlighting possible causes. An assessment of the seriousness of the phenomenon will follow. The latter will be expressed using a number between 1 and 5, where 1 corresponds to minor damage and 5 to very serious damage. The last field (diffusion) quantifies the extent of the damage through a percentage. This value will be defined by calculating the ratio between the damaged area and the healthy area or areas affected by other pathologies. 

Finally, two further entries were planned to link to the model possible graphic representations describing the decay (mapping of the decay or close up photographs). 

### 4.9. Conservation Activities

The group dedicated to the conservation activities is particularly important for the purposes of planned conservation, as it allows operators to easily keep track of the interventions performed on the building.

The first item (recent interventions) requires information to be provided regarding the last maintenance operation carried out on the analyzed element. Using a string of text, the date and type of intervention will be recorded (for example: 1995—cleaning). The second field provides information on the date of the latest inspection, allowing the operator to evaluate whether it is time to schedule a new inspection. In this case also, the datum is set as a text string, in order to enable further specifications to be added. As for the third entry, dedicated to monitoring, an option group has been prepared, offering a multiple choice answer. The aim of this field is to indicate the possible presence of monitoring activities, and, secondly, the nature of such activities: instrumental or visual.

Finally, the last field (notes) provides a string text in which any suggestions or particular needs can be highlighted.

### 4.10. Criticalities

Another tool that directly supports the planning of conservation activities is that which refers to critical issues. In this case also, the structure of the property group was developed starting with the “problem analysis form” provided by the guidelines of 2003 [69]. The first entry concerns possible risk factors. Within this field, the main problems concerning the analyzed element will have to be indicated, taking into account both the intrinsic characteristics of the object itself and its surrounding context. The second property is instead designed to collect the criticalities connected to the mentioned risk factors, thus providing a list of the concrete issues that are likely to occur. For example, if high environmental humidity is indicated among the risk factors, the resulting criticality will be the possibility of a biological attack.

To describe the problem as completely as possible, two further items are provided to specify which areas are to be considered most at risk, as well as which are the elements interacting with the analyzed object. This aspect is rather relevant for preventive purposes, as it enables the possible impact of the damage caused to elements to be estimated; this is for elements that, although somehow connected, are not directly involved. Indeed, in the long run, even these objects could end up suffering because of the identified criticalities. Analyzing the interactions between different objects can therefore help us to understand why certain situations occur, and to identify proper measures that help to limit the propagation of degradation phenomena, once they occur. A final item is dedicated to preventive measures; these are intended to be used as general indications to ensure the effective control of risk factors.

### 4.11. Accessibility

Like the previous property groups, the “accessibility” group is strongly oriented towards the planning of inspection and maintenance activities. Indeed, it provides information regarding the conformation and features of the spaces and objects so that the professionals in charge can choose the most fitting equipment with which to perform specific tasks.

The group is partly organized according to the UNI 11119:2004 standard [71], which describes the preliminary conditions required in order to properly perform an inspection. More specifically, the standard specifies that the wooden elements should be accessible, clean (not covered by dust or other deposits) and sufficiently illuminated. The failure to fulfil or the partial satisfaction of these preconditions may prevent the inspection or limit the quantity and quality of the resulting data. Starting with these assumptions, the following entries have been created:

- accessibility—this indicates whether the analyzed object is accessible or not. Some possible options have been preset through an option group: inaccessible, direct, with scaffolding, with ladder and other;
- visibility—an option group has also been arranged for this field. It will thus be possible to choose among the following: total, partial, null. While the UNI 11119:2004 [71] uses the term “cleanliness”, in this context it was decided that the term “visibility” would be used instead. This was carried out to also include the visibility conditions that are caused by the object’ position. In fact, if the beam—although perfectly clean—should have a surface that adheres to a wall, its overall visibility would only be partial.
- lighting—a multiple selection option group has been set up to describe lighting conditions. It will be possible to alternatively choose one of the following items: absent, natural, artificial. If any light source is present, it will be possible to specify whether it is sufficient or insufficient. The operator will thus be aware of the need to provide lighting when performing conservation activities.

Finally, a further entry (notes) will collect possible notes and specifications.

### 4.12. Historical Documents

Given the large amount of documents gathered throughout the historical analysis, a section has been structured to collect the information that has emerged from archival documents and from the literature. In particular, in order to help retrace the original documents, the first field contains the archival shelf mark. For ease of use, it was decided that a field (“document transcription”) would be added in which the transcription of the document could be accessed via a hypertext link. Should there be any specific bibliographic references, it will be possible to indicate them in the field “bibliographic references”.

This property group could be seen as the continuation of the massive digitization work of the historical archive of the Opera di Santa Maria del Fiore, started by Margaret Haines [75], and continued by the Opera itself [76].

### 4.13. Other

The final group of information provides some general information regarding the compilation of the database and, specifically, the name of the compiler, the date of compilation and possible notes. Since the informative tool is supposed to be constantly fed new information, one last entry has been provided; this can be used to indicate the dates of later updates (or revisions), together with the names of the operators involved.

### 4.14. Priority Index

In addition to the mentioned property groups, a further section dedicated to the definition of an intervention priority index has been established. The latter represents an extremely relevant tool for the planning of conservation activities, as it enables the existing critical issues to be quickly identified and operated on in a targeted manner, thus ensuring the better optimization of the available resources [77].

Following the in-depth analysis of the wooden chain, the study turned to the identification of those aspects that are crucial in order to define an effective prevention strategy. By attributing a score to each of the mentioned aspects, it will be possible to determine, by means of simple algorithms, the sought priority index. In particular, the latter results from the sum of the scores attributed to the following macro categories: historical–architectural value, damage conditions and risk conditions. Since the rating scale ranges from 0 to 5, the index will be expressed using a number between 0 and 15, where 15 indicates the highest priority and 0 the lowest priority.

In turn, the macro categories’ evaluations will be calculated as the average between the scores of some sub-categories, including age and quality of workmanship (for the historical–architectural value), material decay and loss of resistance (for damage conditions), and inspection period and criticalities (for the risk conditions).

Each sub-category will be graded with a score from 0 to 5, where 0 indicates an optimal situation and 5 a situation of maximum seriousness. Table 2 briefly describes the criteria adopted to define the intervention priorities. The proposed criteria can be recalibrated and refined after testing their efficiency. In particular, they will need to satisfy any requests from the technical office of the Opera.

As for the compilation of the information model, the data will be filled in as follows:

- for sub-categories, the score must be entered manually by selecting one of the preset values included in the option group. The operator in charge will therefore be able to easily choose a value between 0 and 5 from a drop-down menu. For the sake of clarity, a brief on the method of compilation will be offered within the description of the property.
- For macro-categories, by using the “expression” function, it is possible to automatize the calculation. The software will thus automatically calculate the average between the scores attributed to the sub-categories.
- For the priority index, the value of the priority index will also be calculated automatically by the program. The “expression” function will operate the overall sum of the scores related to the historical–architectural value, the damage conditions and the risk conditions.

## 5. Discussion

Ultimately, the aim of the proposed HBIM model is not merely to offer a precise representation of cultural heritage, but also to effectively support its conservation [78]. The objective that was pursued in the development of the information model offers a widely applicable method which, through the systematization and exploitation of information, can offer concrete support to the planning and execution of conservation activities. In particular, the operational contribution of the proposed method can be summarized in the following advantages:

- Data usability: the fact that information is collected and organized within a single model will facilitate the knowledge process, which is at the basis of conservation: the greater the availability of data, the better the planning of inspection and maintenance activities, both in terms of the timing and methods to be used.
- Intervention priorities: the priority indexes, highlighting the most critical issues, can be easily translated into a list of interventions to be carried out. The scheduling will reflect the urgency expressed by the index, resulting in farsighted planning and in the optimization of available resources, which will be firstly allocated to non-derogable activities.
- Pocket archive: the possibility of consulting the model in situ, via portable devices, will allow professionals in charge of conservation activities to consult the available data and use them to carry out better targeted and more exhaustive analyses.

Whilst the information model has been developed on an experimental basis for the wooden chain, the hope is that in the future it will expand to include data concerning the whole dome. In particular, the developed tool could be particularly useful for the mapping of the extrados of the dome, carried out on a six-monthly basis. On this occasion, the operators could benefit from consulting the database and could verify in real time the timeline of the interventions, as well as the possible presence of specific issues. Similarly, the model could support the preservation of the intrados of the dome, with particular reference to the frescos. Restorations occurred in the 1990′s have in fact produced a lot of data, which could be fruitfully included in the informative model [79].

The most substantial advantages could, however, derive from the georeferencing of the 166 instruments that make up the complex structural monitoring system of the Dome [80]. This will enable information to be associated with each device, such as Excel files, graphs describing crack propagation, technical specifications on the monitoring devices, photographic surveys, as well as any other reference that could be useful for a better assessment of the crack pattern. As a principle, if the information model was to be applied to the entire dome (or even to the entire cathedral), the validity of the proposed method would further increase. An overview of the whole structure would in fact enable, in addition to the individual elements, their reciprocal interactions to be considered, identifying the possible risks caused by those very interactions. Positive effects could also be recorded in terms of resource optimization; activities to be carried out on elements that are close to one another could be synchronized, so as to reduce the overall number of inspections, while obtaining the same results.

Besides these potentials, the proposed method, however, shows some limitations that could be overcome in future steps of the research. First of all, the presented solution currently lacks a specific application with which to record information while on-site. Indeed, the ArchiCAD BIMx application, available for portable devices, allows one to access the model while on site, but not to feed data into it in real time. This is a rather crucial aspect and could be fixed with the collaboration of ArchiCAD developers. Another relevant limitation is that the articulation of information only concerns wooden and iron elements. While property groups referring to wooden beams could easily be applied (with due adjustments) to other case studies, no property groups have been defined for masonry objects, nor for many other types of objects. At this stage, it would thus only be possible to apply the proposed method to wood carpentry or wooden artifacts in general. The setting up of property groups for other elements will broaden the field of application. Finally, while the idea of using a simplified model is at the base of the proposed strategy, in the specific case, the 3D model could be improved. To this end, a more recent and technologically advanced survey of the dome should be used and the LOD could be increased to 300, so as to have a simplified but more reliable geometry.

The assessment of the accuracy needed for the 3D model should, however, also take into account the opportunity of using the model to perform structural analyses. The research is ongoing, and is now focusing on the definition of the FEM numerical translation of the entire model of the dome. The interoperability between BIM and FEM is one of the frontiers of HBIM research [81,82,83,84,85]. In this regard, one of the questions to address is whether the same model should be used for HBIM and finite element analysis. Indeed, the level of detail required to design restoration interventions is usually higher than the one needed for structural analysis. In this second case, it is essential that irregularities that can influence the mechanical behavior and small defects that are not relevant from a structural point of view are distinguished between [81]. It is also important to understand whether and how other qualitative data could be exported together with the geometry (e.g., stratigraphic units). The analyzed case studies show that most of the time, the model used for the finite element analyses has the same level of detail as the BIM [81,84]. However, other solutions are also proposed [85]. This phase of the research will surely highlight further criticalities that will help to enhance this first draft of the HBIM.

The validation of the presented method can, however, only derive from its application, primarily to the wooden chain. It will only be possible to verify its effectiveness once it is evaluated by the Opera of Santa Maria del Fiore, highlighting any critical issues and suggestions for its refinement.

## 6. Conclusions

The need to carry out an in-depth historical analysis prior to any intervention on historical heritage is by no means new. Indeed, the idea that historical investigation is a primary moment of the restoration process [1] has been long and widely acknowledged.

Taking Brunelleschi’s wooden chain as a pretext, the research ultimately aims to retrace the stages of the knowledge process, which, from the very first approach to the monument, leads to its conservation. This process involves many tools: some traditional, some innovative, some specifically intended for restoration, and some others borrowed from other scientific sectors. In such a complex context, the search for balance between different instances represents a critical node [86]. This study therefore reflects an attempt to propose a method that brings conservation back to the center, which is the ultimate objective towards which any analysis and tools should be addressed. The concept is well expressed by Marco Dezzi Bardeschi, who, at the end of the last century, stated that:

“[…] today it is no longer possible to speak of restoration if not identifying it with the primary requirement of conservation, i.e., if it is not intended erga omnes as the exclusive cultural and technical commitment to ensure above all the persistence and effective permanence of the asset. The mutable series of notions of “value” that every historical moment and every subject cannot fail to express with regard to built heritage, today can only serve to define a degree or a scale of priorities to be attributed to the conservation intervention […].”

[87]

By proposing an information model for the wooden chain, the information collected and analyzed throughout the knowledge process will come to constitute the basis for the definition of an intervention priority index. The latter will facilitate the scheduling of conservation activities, optimizing, at the same time, the available resources (also economic). This idea is perfectly in line with the current preservation framework, with particular reference to the Italian Code of Cultural Heritage and Landscape, which states that

“The conservation of the cultural heritage is ensured by means of a consistent, coordinated and programmed activity of study, prevention, maintenance and restoration.”

[88]

On the other hand, decree n.312/2021 introduced a timeline by which to gradually make BIM mandatory in the case of public contracts of over 1 million in value. By 2025, this will regard new constructions as well as built heritage. The combination of planned conservation and HBIM thus appears to be fruitful and well suited to the upcoming changes in regulations. 

Ultimately, it is in the management of information and its operative exploitation that the present study identifies a modus operandi, in the awareness that—regardless of the adopted technology—knowledge continues (and will continue) to play a primary role in conservation.

## Figures and Tables

**Table 1:** The codification system developed to univocally name the elements comprising the dome of Santa Maria del Fiore.

CUPOLA OF SANTA MARIA DEL FIORE (Cu)							
Sector 1 **S1**	Drum **T**						**Cu. S1.T**
	Outer shell **Ce**	Intrados **int**					**Cu.S1.Ce_int**
		Extrados **est**					**Cu.S1.Ce_est**
	Inner shell **Ci**	Intrados **int**					**Cu.S1.Ci_int**
		Extrados **est**					**Cu.S1.Ci_est**
	Ribs **C**	Corner **a**					**Cu.S1-2.Ca**
		Central **m**	**1**				**Cu.S1.Cm1**
			**2**				**Cu.S1.Cm2**
	Cavity **I**	Room 1 **V1**	Level 1 **L1**	Wooden chain **Cl**	Beam **T**	**1**	**Cu.S1.I_V1.L1.Cl.T1**
						**2**	**Cu.S1.I_V1.L1.Cl.T2**
						…	
					Joint **G**	**1**	**Cu.S1.I_V1.L1.Cl.G1**
						**2**	**Cu.S1.I_V1.L1.Cl.G2**
						…	
			Level 2 **L2**				**Cu.S1.I_V1.L2**
			Level 3 **L3**				**Cu.S1.I_V1.L3**
		Room 2 **V2**	Level 1 **L1**	Wooden chain **Cl**	Beam **T**	**1**	**Cu.S1.I_V2.L1.Cl.T1**
						**2**	**Cu.S1.I_V2.L1.Cl.T2**
						…	
					Joint **G**	**1**	**Cu.S1.I_V2.L1.Cl.G1**
						**2**	**Cu.S1.I_V2.L1.Cl.G2**
						…	
			Level 2 **L2**				**Cu.S1.I_V2.L2**
			Level 3 **L3**				**Cu.S1.I_V2.L3**
		Room 3 **V3**	...				

**Table 2:** Brief description of the criteria used to define the intervention priority index.

Priority	N°
The priority index is expressed through a number between 0 and 15. It results from the sum between the scores attributed to the historical-architectural value (0 to 5 points), to the damage conditions (0 to 5 points) and to the risk conditions (0 to 5 points). The index quickly highlights the existing criticalities, thus enabling to establish proper intervention priorities.	
**Historical-Architectural Value**	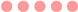
The rating for the historical-architectural value is calculated as the average between the score assigned to the historical significance (dating) and that given to the quality of workmanship. Each sub-field is evaluated using a number ranging from 0 to 5.	
*Dating*	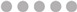
The score evaluates the historical significance of the object. The highest value (5) is attributed to original elements; the lowest value (0) is assigned to the most recent additions and/or additions of little historical interest.	
*Quality of workmanship*	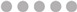
Assessment of the technical and qualitative value of the object taking into account the manufacturing of individual elements and the techniques used to assemble them.	
**Damage Conditions**	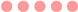
Overall evaluation of the conditions of the element, considering both the material decay (state of repair) and the possible loss of efficacy of the element (or system) comparing to its original state. Each sub-field will be assigned a number ranging from 0 to 5. The average between the latter will define the overall score of the section “Damage conditions”	
*State of repair*	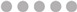
The score reflects the state of conservation of the elements, with particular reference to the decay of the materials.	
*Loss of efficacy*	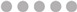
The index expresses an evaluation concerning the possible loss of efficacy of the element (residual mechanical properties). This evaluation can be made either basing on instrumental analyses and/or visual inspections	
**Risk Condition**	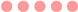
Overall assessment of the existing risks defined as the average between the inspection period (0 to 5 point) and criticalities (0 to 5 points).	
*Inspection period*	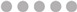
The index evaluates the length of time since the last inspection or conservation activity took place. Considering a one-year period as optimal, the minimum score (0) will be assigned if the last inspection occurred within the last year, while the maximum score (5) will be assigned if the last inspection occurred more than five years ago.	
*Criticalities*	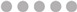
The score evaluates the intrinsic and estrinsic criticalities concerning the element/object and its surrounding environment, also taking into account all the possible interactions with other elements.	

## Data Availability

Data is contained within the article.
